# Identifying Potential Biomarkers of Prognostic Value in Colorectal Cancer *via* Tumor Microenvironment Data Mining

**DOI:** 10.3389/fgene.2021.787208

**Published:** 2022-02-03

**Authors:** Lei Li, Xiao Du, Guangyi Fan

**Affiliations:** ^1^ College of Life Sciences, University of Chinese Academy of Sciences, Beijing, China; ^2^ BGI-Qingdao, BGI-Shenzhen, Qingdao, China; ^3^ BGI-Shenzhen, Shenzhen, China

**Keywords:** immune, stromal, hub genes, colorectal cancer, survival analysis, tumor location

## Abstract

Colorectal cancer (CRC) is a common cancer that has increased rapidly worldwide in the past decades with a relatively high mortality rate. An increasing body of evidence has highlighted the importance of infiltrating immune and stromal cells in CRC. In this study, based on gene expression data of CRC patients in TCGA database we evaluated immune and stromal scores in tumor microenvironment using ESTIMATE method. Results showed there was potential correlation between these scores and the prognosis, and that patients with higher immune score and lower stromal score had longer survival time. We found that immune score was correlated with clinical characteristics including tumor location, tumor stage, and survival time. Specifically, the right-sided colon cancer had markedly elevated immune score, compared to left-sided colon cancer and rectal cancer. These results might be useful for understanding tumor microenvironment in colorectal cancer. Through the differential analysis we got a list of genes significantly associated with immune and stromal scores. Gene Set Enrichment and protein-protein interaction network analysis were used to further illustrate these differentially expressed genes. Finally, 15 hub genes were identified, and three (CXCL9, CXCL10 and SELL) of them were validated with favorable outcomes in CRC patients. Our result suggested that these tumor microenvironment related genes might be potential biomarkers for the prognosis of CRC.

## Introduction

Colorectal cancer (CRC) is one of the most commonly occurring cancers, whose incidence occupies 10% of all cancer diagnoses (Sung et al., 2021; Wong et al., 2021). As the second most common cause of cancer death, Colorectal cancer has been increasing rapidly in the past decades with over 1.9 million new cases reported in 2020 (Arnold et al., 2017; Sawicki et al., 2021). Colorectal cancer may develop on either the proximal colon (right side), the distal colon (left side) or the rectum. Right-sided colon cancer (RCC) differs from the left-sided colon cancer (LCC) and rectal cancer (RC) in pathogenesis and prognosis, exhibiting distinct molecular characteristics and histology (Baran et al., 2018; Imperial et al., 2018; Siegel et al., 2020). Presently, CRC screening is not common and the diagnosis is usually made after the onset of symptoms. Because the tumor status and TNM stage at diagnosis have a fundamental role in CRC prognosis, early symptom investigation and diagnosis are of high importance (Bosch et al., 2011; Kawakami et al., 2015). However, although CRC prevalence is high, the awareness of colorectal cancer and its symptoms is relatively low. Due to wide variation in colorectal cancer and complexity in treatment outcome prediction, investigation for new strategies and new biomarkers is necessary in CRC for improving prognosis.

It has been documented that tumor microenvironment (TME) has a great impact on tumor cells and clinical outcomes (Turley et al., 2015; Lim et al., 2018). Apart from tumor cells, TME also comprises a variety of nontumor components including endothelial cells, immune cells, inflammatory mediators, and extracellular matrix (ECM) molecules (Lorusso and Rüegg, 2008; Bolouri, 2015). The cells and molecules in the TME are in a dynamic process, jointly promoting tumor immune escape, tumor growth and metastasis (Quail and Joyce, 2013). Accumulating evidence suggests that the stromal and immune cells, which constitute two main nontumor components in the TME, are valuable in investigating tumor diagnosis and clinical outcome (Kalluri and Zeisberg, 2006; Hanahan and Weinberg, 2011; Fridman et al., 2012). Recent evidence has indicated that tumor microenvironment plays a significant role in colorectal carcinogenesis, metastasis and the choosing of therapy strategies (Peddareddigari et al., 2010; Pedrosa et al., 2019). T cells, a major part of the immune system, were described to be of major importance for tumor growth, invasion, early metastasis and prognosis in colorectal cancer (Pagès et al., 2005; Mlecnik et al., 2011). Calon et al. suggested that high expression of mesenchymal genes associated with poor outcomes in CRC patients is primarily caused by stromal cells instead of epithelial cancer cells (Calon et al., 2015). To promote the understanding of cancer prognosis, efforts have been made in studying tumor microenvironment components and developing novel immunotherapeutic strategies in recent years. Algorithms such as ESTIMATE (Estimation of STromal and Immune cells in MAlignant Tumor tissues using Expression data) (Yoshihara et al., 2013) have been developed to predict tumor purity and levels of infiltrating stromal and immune cells in tumor, such as gastric cancer, hepatocellular carcinoma and colorectal cancer (Mao et al., 2018; Deng et al., 2019; Wang et al., 2019).

To promote the understanding of CRC microenvironment and prognosis, in this study we took use of ESTIMATE algorithm and public database to evaluate the tumor-infiltrating immune and stromal cells of TME. By performing survival analysis and correlation analysis, we explored the relationship between immune/stromal score and clinical factors in CRC. Moreover, we aim to extract a list of tumor microenvironment associated genes of prognostic value, through the differential analysis, network construction and survival analysis. We hope to provide insights to investigate stromal and immune cells of CRC and offer evidence to potential prognostic markers.

## Materials and Methods

### Data Collection and Preprocessing

In this study, gene expression profiles of colorectal cancer were downloaded and collected from The Cancer Genome Atlas (TCGA) data portal (https://portal.gdc.cancer.gov/) using TCGAbiolinks (Colaprico et al., 2016) R package. Relevant clinical information including age, gender, survival time, pathologic stage, and tissue or organ of origin were also obtained from TCGA database. Patients with primary tumor expression and survival information were retained in this study. Before further analysis, TCGA gene expression profiles were normalized using R package DESeq2 (Love et al., 2014).

GSE41258 expression and clinical data were also downloaded from the Gene Expression Omnibus (GEO) database as the validation set. The GSE41258 dataset was processed *via* the Affymetrix MAS5 background correction algorithm using affy package (Gautier et al., 2004) in R and log2 transformation. Probe sets were transformed into gene sets by retaining only the probes with the highest expression levels if one gene corresponds to multiple probes. When multiple genes per probe, this probe would be discarded.

### Estimation of Immune and Stromal Scores

The normalized expression data was analyzed by the ESTIMATE algorithm for calculating the Immune and Stromal Score. We used ESTIMATE to calculate the fraction of immune and stromal cells in tumor using the gene expression data. In our study v.1.0.13 estimate R package (Yoshihara et al., 2013) was used to predict the level of infiltrating immune cells (immune score) and the level of infiltrating stromal cells (stromal score).

### Survival Analysis Based on Immune and Stromal Scores

Survival analysis was performed by R package survival (Therneau, 2019) and survminer (Kassambara et al., 2019) to assess the association of immune and stromal score with prognosis. The best cut-off value of immune/stromal score was inferred using R program surv_cutpoint. Subsequently, patients were divided into two groups (high *vs*. low) based on the cut-off value. The Kaplan-Meier (KM) method was used to estimate the likelihood of survival based on the observed overall survival time. Overall Survival differences between high and low score groups were compared by log-rank test.

### Differential Gene Expression Analysis

We analyzed differentially expressed genes (DEGs) between high score and low score groups using R package DESeq2 (Love et al., 2014), which based on the negative binomial distribution algorithm. And |log2 fold change (FC)| > 2 and *p* value < 0.01 were selected as the criteria to select the significantly different genes. R package pheatmap (Kolde, 2019) was used to visualize the DEGs.

### Function Analysis

To explore the potential function of DEGs, function analysis was carried out by using the Gene Set Enrichment Analysis (GSEA) web server (Mootha et al., 2003; Subramanian et al., 2005). Enrichment analyses of hallmark gene sets, ontology gene terms (cellular component, molecular function, and biological process), and KEGG gene sets were selected to extract biological insight in different risk groups. The top 20 biological functional terms with False discovery rate (FDR) q-value below 0.01 were selected.

### PPI Network Construction and Hub Gene Selection

To further investigate the relationship between different genes, the protein-protein interaction (PPI) network analysis was performed *via* the version 11.5 STRING (Search Tool for the Retrieval of Interacting Genes, https://string-db.org/) (Szklarczyk et al., 2015), an online tool and database of protein-protein interaction. A minimum required interaction score > 0.7 were selected and reconstructed in the Cytoscape (Shannon et al., 2003) software. In a gene candidate module, one gene with high correlation with other genes is called a hub gene. In this study, We used CytoHubba plugin (Chin et al., 2014) in Cytoscape v3.7.1 to find hub genes in PPI network. The top 15 genes with the highest prediction scores calculated by the Maximal Clique Centrality (MCC) algorithm were defined as the hub genes.

### Statistical Analysis

All statistical analyses were performed in R statistical environment version ≥ 3.5.0. Cox proportional hazard regression survival analysis was applied to overall survival time with different clinical features including age, gender, tumor location, tumor stage, immune score and stromal score. Correlations between the clinical factors and immune/stromal score were also calculated in this study. Kruskal-Wallis Test for three or more groups and Wilcoxon Test for two groups were used to estimate the P value.

## Results

### Tumor Immune and Stromal Scores Significantly Associated With Prognosis in CRC

HTSeq-Counts based gene expression profiles and clinical information of 613 CRC patients were downloaded from TCGA database. In this cohort, patients were diagnosed with colorectal cancer between 1998 and 2013 and their sequencing and clinical information were collected into the TCGA database. Among them, 286 (46.7%) patients were female and 327 (53.3%) patients were male. The ages ranged from 31 to 90. Clinical diagnosis included 189 (30.8%) cases with right-sided colon cancer, 132 (21.5%) cases with left-side colon cancer, and 85 (13.9%) cases with rectal cancer. The Pathologic stage I, stage II, stage III and stage IV accounted for 16.8% (*n* = 103), 37.0% (*n* = 227), 28.9% (*n* = 177) and 14.0% (*n* = 86) of the total number (Table 1). In addition, based on ESTIMATE algorithm immune and stromal scores were obtained. Stromal scores for the analyzed CRC cohort ranged from -2,531.36 to 1,481.74, and immune scores were distributed between −1,724.23 and 1,856.93, respectively. The average immune score was −600.92 and the median was −658.63. The average stromal score was −966.83 and the median was −1,026.69.

**TABLE 1:** Summary and Cox Regression Analysis of overall survival for TCGA CRC study dataset.

Characteristics	Count	Univariate Cox		Multivariate Cox	
		Hazard ratio (95% CI)	P-value	Hazard ratio (95% CI)	P-value
**Age**	613	1.03 (1.015-1.049)	<0.001	1.04 (1.021-1.067)	<0.001
**Gender**				
Female	286	1	-	1	-
Male	327	1.02 (0.710-1.454)	0.93	0.88 (0.554-1.400)	0.59
**Location**				
Right	189	1	-	1	-
Left	132	0.70 (0.435-1.134)	0.15	0.58 (0.349-0.974)	0.04
Rectum	85	0.72 (0.386-1.326)	0.29	0.55 (0.279-1.094)	0.09
**Stage**				
Stage I	103	1	-	1	-
Stage II	227	1.72 (0.712-4.150)	0.23	1.03 (0.384-2.775)	0.95
Stage III	177	3.19 (1.345-7.580)	0.01	2.31 (0.870-6.151)	0.09
Stage IV	86	8.62 (3.647-20.370)	<0.001	7.36 (2.803-19.327)	<0.001
**Stromal score**				
High	230	1	-	1	-
Low	383	0.69 (0.483-0.998)	0.05	0.66 (0.332-1.312)	0.24
**Immune score**				
High	425	1	-	1	-
Low	188	1.44 (1.001-2.071)	0.05	2.07 (1.060-4.043)	0.03

**FIGURE 1:**
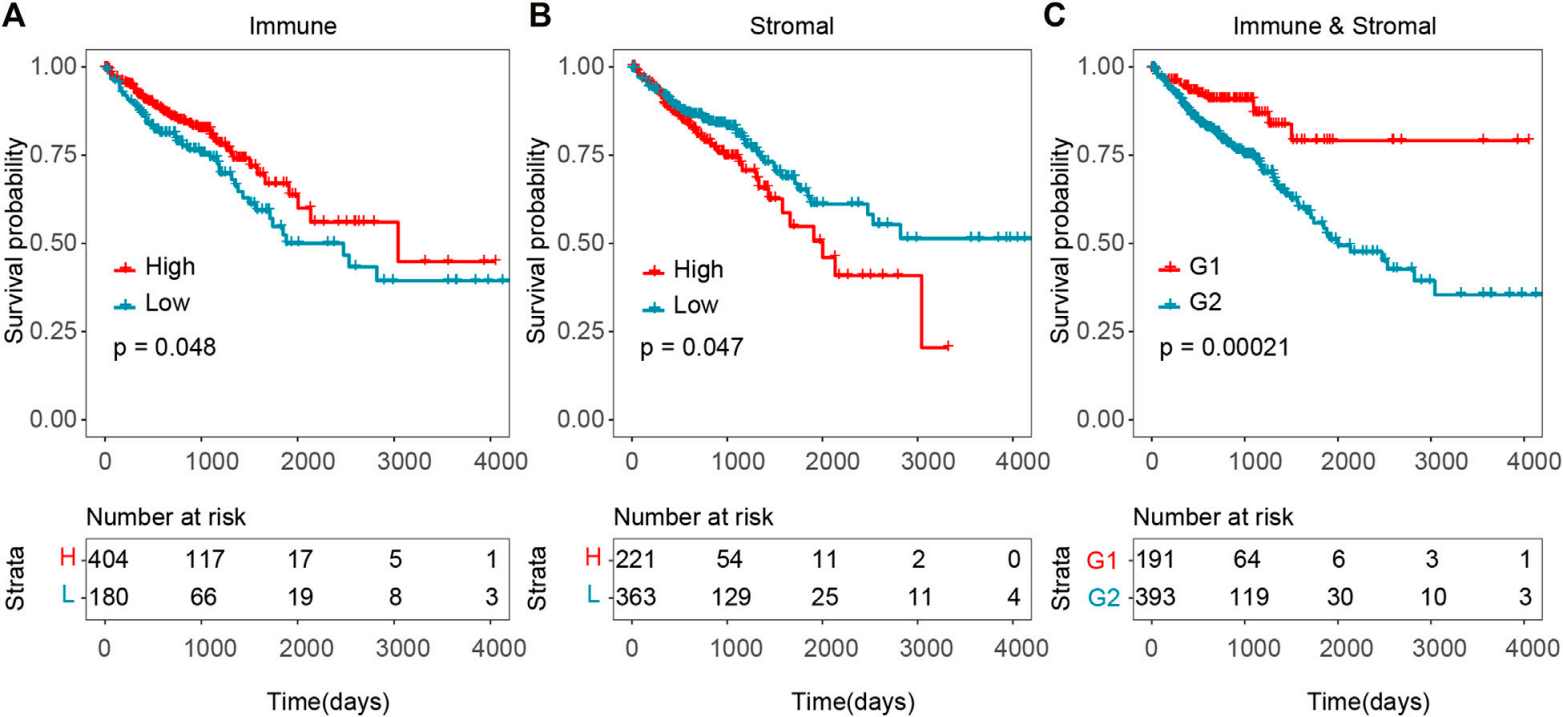
Association between tumor microenvironment and overall survival time in TCGA CRC cohort. **(A)** Kaplan–Meier curves of high and low immune score groups. **(B)** Kaplan–Meier curves of high and low stromal score groups. **(C)** Kaplan–Meier curves of G1 (high immune score and low stromal score group) *versus* G2 (low immune and stromal score group, high immune and stromal score group, low immune score and high stromal score group).

To explore the potential correlation of overall survival time with stromal and immune scores, 613 CRC cases were divided into high- and low-score groups according to the cut-off of stromal/immune scores. As shown in Figure 1, survival analysis indicated that both the immune and stromal scores were significantly correlated with overall survival time, and that patients with high immune score or low stromal score significantly correlated with better overall survival time (Figures 1A,B, *p*-value = 0.048 for immune score and p-value = 0.047 for stromal score, log-rank test). Patients with high immune score had a median overall survival time of 101.4 months, while patients with low immune score had a median survival of 62.7 months. Patients with lower stromal score also had a longer median overall survival compared to those with high stromal score. Especially, patients with combined high immune score and low stromal score have a significantly better overall survival time than others (Figure 1C, p-value = 0.00021, log-rank test).

In order to validate these results which were obtained from the TCGA database, we downloaded and analyzed another independent cohort in GEO database. We retrieved 182 CRC patients’ gene expression data and clinical information from GSE41258 cohort as validation set. Although the difference was not statistically significant, Patients with high immune score displayed a longer median survival (Supplementary Figure S1A, high- vs. low-score = 91 *vs*. 86 months). And patients with lower stromal score showed a longer median survival (Supplementary Figure S1B, high- *vs*. low-score = 72 *vs*. 113 months). Consistently, patients with high immune score and low stromal score in the validation cohort had a longer survival time (Supplementary Figure S1C, p-value = 0.021, log-rank test). These results indicated that higher level of immune score and lower level of stromal score in CRC might mean the favorable survival outcome, which might provide potential prognosis stratification factors for clinical predictions.

### Immune Scores Correlated With Tumor Location and Tumor Stage in CRC

**FIGURE 2:**
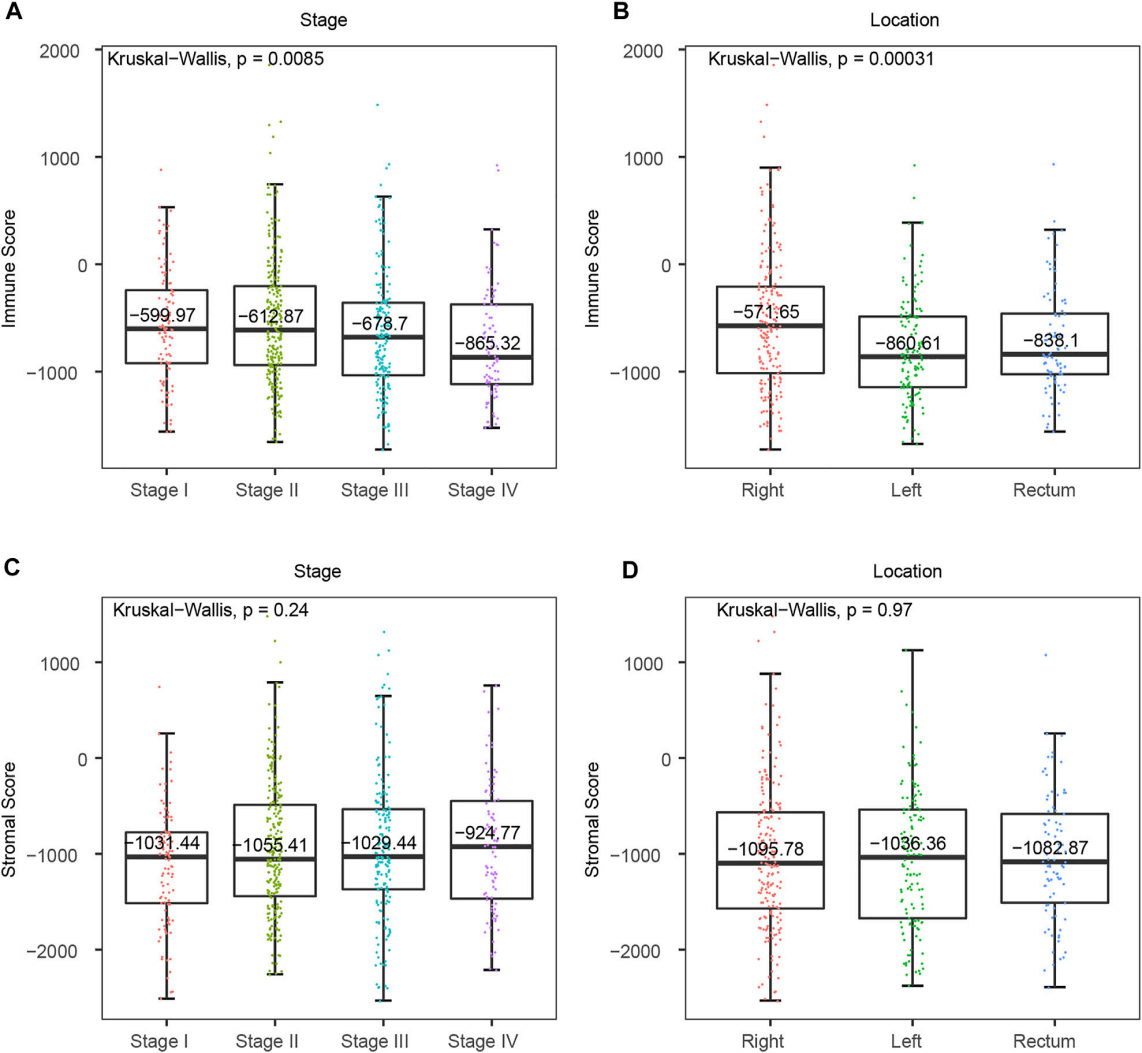
Association between tumor microenvironment scores and clinical features in TCGA CRC cohort. **(A)** Distribution of immune scores in consecutive CRC tumor stages. **(B)** Distribution of immune scores from distinct CRC primary tumor locations. **(C)** Distribution of stromal scores in consecutive CRC tumor stages. **(D)** Distribution of stromal scores from different CRC primary tumor locations.

To determine the clinical significance of immune and stromal scores, we investigated the association between immune/stromal score and clinical features, and the results suggested that the right-sided colon cancer have a significantly higher immune score. Immune score significantly correlated with tumor stage and tumor location (Figures 2A,B, p-value < 0.01). The median immune score of the RCC patients ranked the highest of all three tumor location subtypes, and the LCC subtype cases had the lowest immune scores (RCC *vs* RC = −571.65 *vs* −838.1, p-value = 0.019, RCC *vs* LCC = −571.65 *vs* −860.61, p-value = 0.00012, LCC *vs* RC = −860.61 *vs* −838.1, p-value = 0.23, Wilcoxon Test) (Figure 2B). Similarly, the rank order of immune scores across tumor stage from highest to lowest was Stage I > Stage II > Stage III > Stage IV (Figure 2A). What’s more, we found immune score was also significantly associated with tumor location and the RCC also had the highest immune score in GSE41258 dataset (Supplementary Figure S2B, p-value = 0.032), which indicated that immune score might be predictive in the classification of CRC tumor location. However, we found no significant differences between stromal scores with CRC tumor stage or location (Figures 2C,D, p-value > 0.05). Consequently, immunotherapy is likely to be more effective for right-sided colon cancer with more immune infiltration and activation in CRC.

### Differential Expressed Genes Revealed by Immune and Stromal Scores in CRC

**FIGURE 3:**
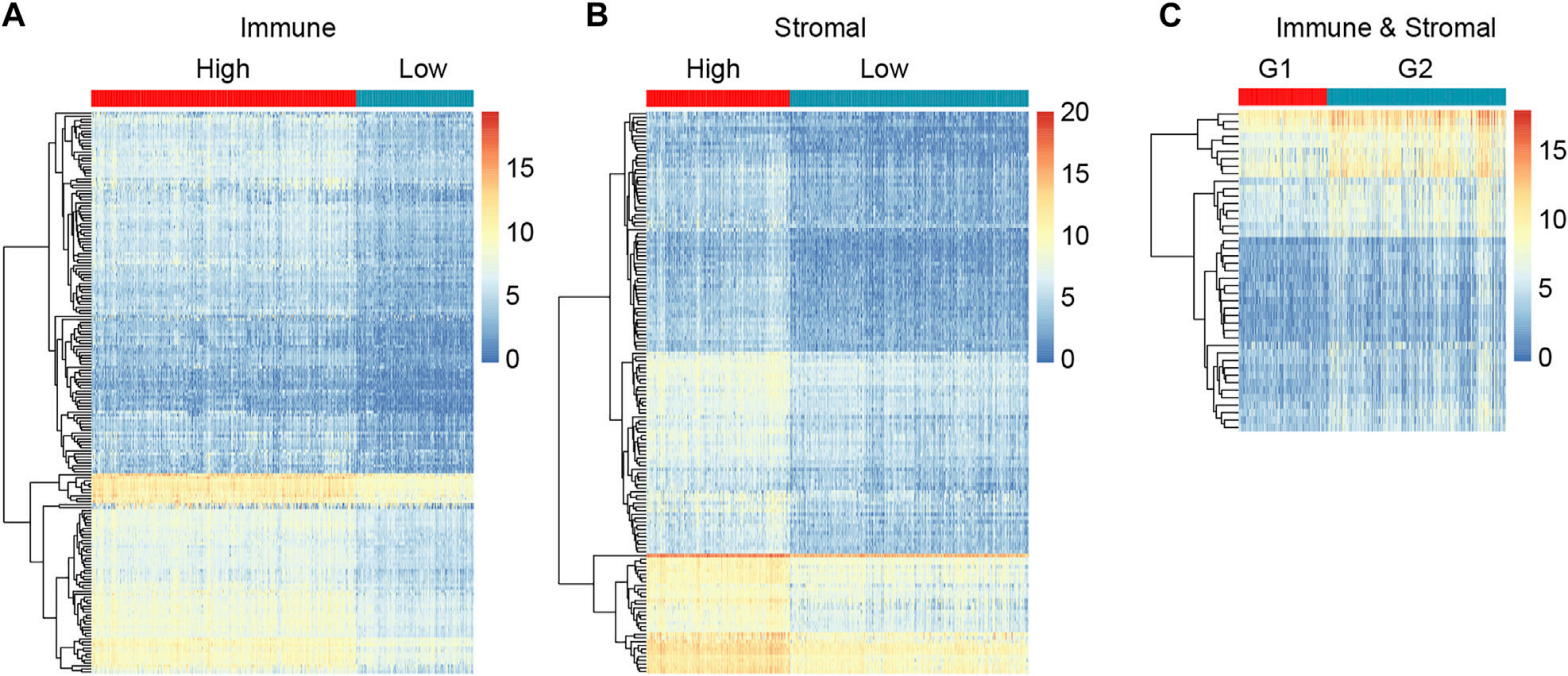
Compare of gene expression profiles in different immune/stromal score groups to identify DEGs. **(A)** Heatmap showing DEGs in high *versus* low Immune score groups. **(B)** Heatmap showing DEGs in high *versus* low Stromal score groups. **(C)** Heatmap showing DEGs in G1 (high immune score and low stromal score group) *versus* G2 (low immune and stromal score group, high immune and stromal score group, low immune score and high stromal score group).

To reveal the correlation of gene expression profiles with immune and stromal scores, we performed differential expression genes analysis using DESeq2, and 318 DEGs were screened out in total. By comparing immune scores between high- and low-score groups, 188 genes were identified to be differentially expressed genes. A total of 150 DEGs were found for high stromal score as compared to low stromal score. What’s more, we got 43 DEGs when high immune and low stromal score patients were compared to the rest. The expression level of the DEGs in each group was displayed in heatmap (Figure 3). The subsequent analysis in our study were based on these DEGs.

**FIGURE 4:**
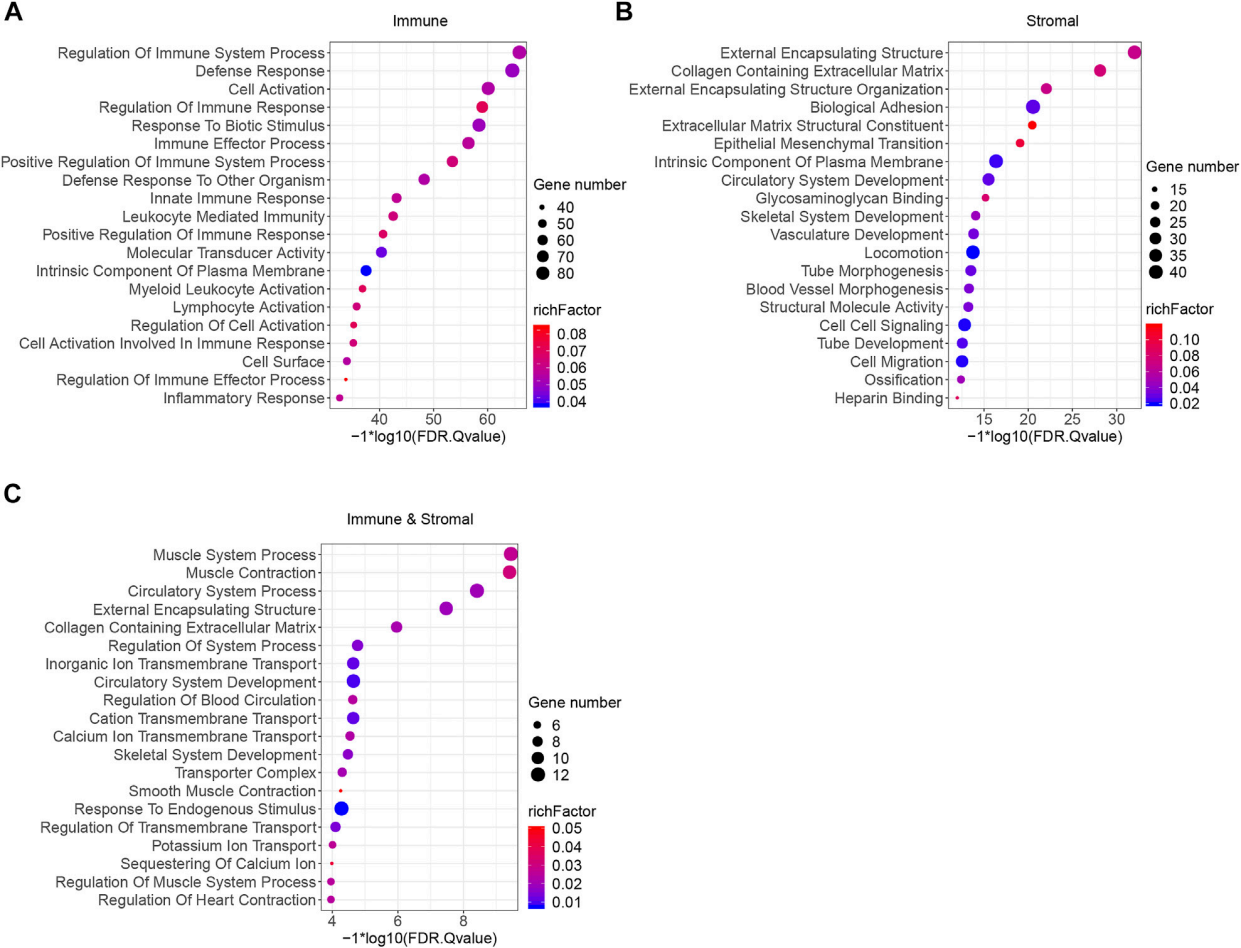
Functional enrichment analysis of immune and stromal scores related DEGs. **(A)** GSEA analysis results of immune score related DEGs. **(B)** GSEA analysis results of stromal score related DEGs. **(C)** GSEA analysis results of immune and stromal score related DEGs. Top 20 terms were exhibited according to the significance of FDR q-value.

To better understand the potential biological functions and mechanisms of DEGs in different immune and stromal score groups, Gene Set Enrichment Analysis was used to annotate the biological roles of these DEGs. GO: BP, GO: CC, GO: MF, KEGG pathways and hallmark gene sets were included in the functional enrichment analysis. The top 20 functional terms of DEGs in each group were shown in Figure 4. For the immune score group, the DEGs were mostly enriched in the regulation of immune system process and defense response. For the stromal score group and combined group, the top biological terms were external encapsulating structure and muscle system process. Moreover, circulatory system development, collagen containing extracellular matrix, external encapsulating structure, intrinsic component of plasma membrane, and skeletal system development were enriched in at least 2 groups. According to the result of GSEA, it could be concluded that these 318 DEGs were mostly involved in the immune regulation biological process that modulates the frequency, rate, extent of an immune system process, and cytokine-cytokine receptor interaction pathway.

### Hub Gene Selection Based on PPI Network

**FIGURE 5:**
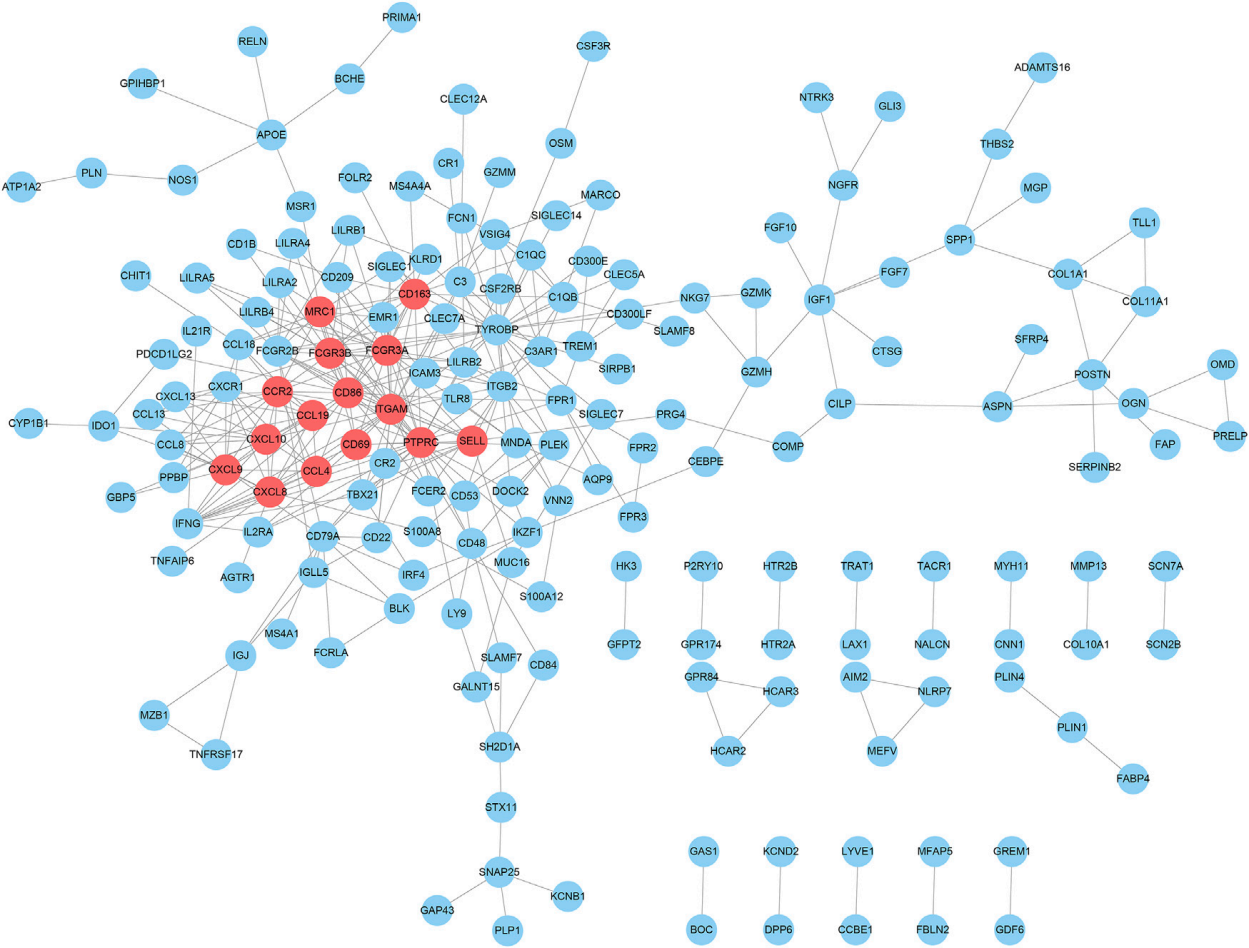
PPI network analysis of DEGs and their hub genes screen. The hub gene nodes were highlighted in red.

In order to evaluate the protein interactive relationships among DEGs, PPI network was constructed based on STRING database and nodes that reported high scores in the network were screened as hub genes. A total of 318 differential expressed genes comprised 318 nodes and 372 edges based on STRING database, and result was visualized in Figure 5 after hided disconnected nodes in the network. Following STRING analysis, the network was reconstructed in the Cytoscape. According to the calculation of CytoHubba plugin module, we identified a list of important genes, from which the top fifteen genes identified by the MCC algorithm were used for further analysis. Finally, 15 genes were selected as hub genes (CD86, ITGAM, PTPRC, FCGR3A, FCGR3B, MRC1, CD163, CCR2, SELL, CD69, CXCL10, CXCL8, CXCL9, CCL19 and CCL4), which were marked with red color in the PPI network (Figure 5). And we found that these genes were significantly enriched in the external side of plasma membrane, cell surface and chemokine receptor binding according to Gene Set Enrichment Analysis (Supplementary Table S1).

### Identification and Validation of Survival Related Hub Genes

**FIGURE 6:**
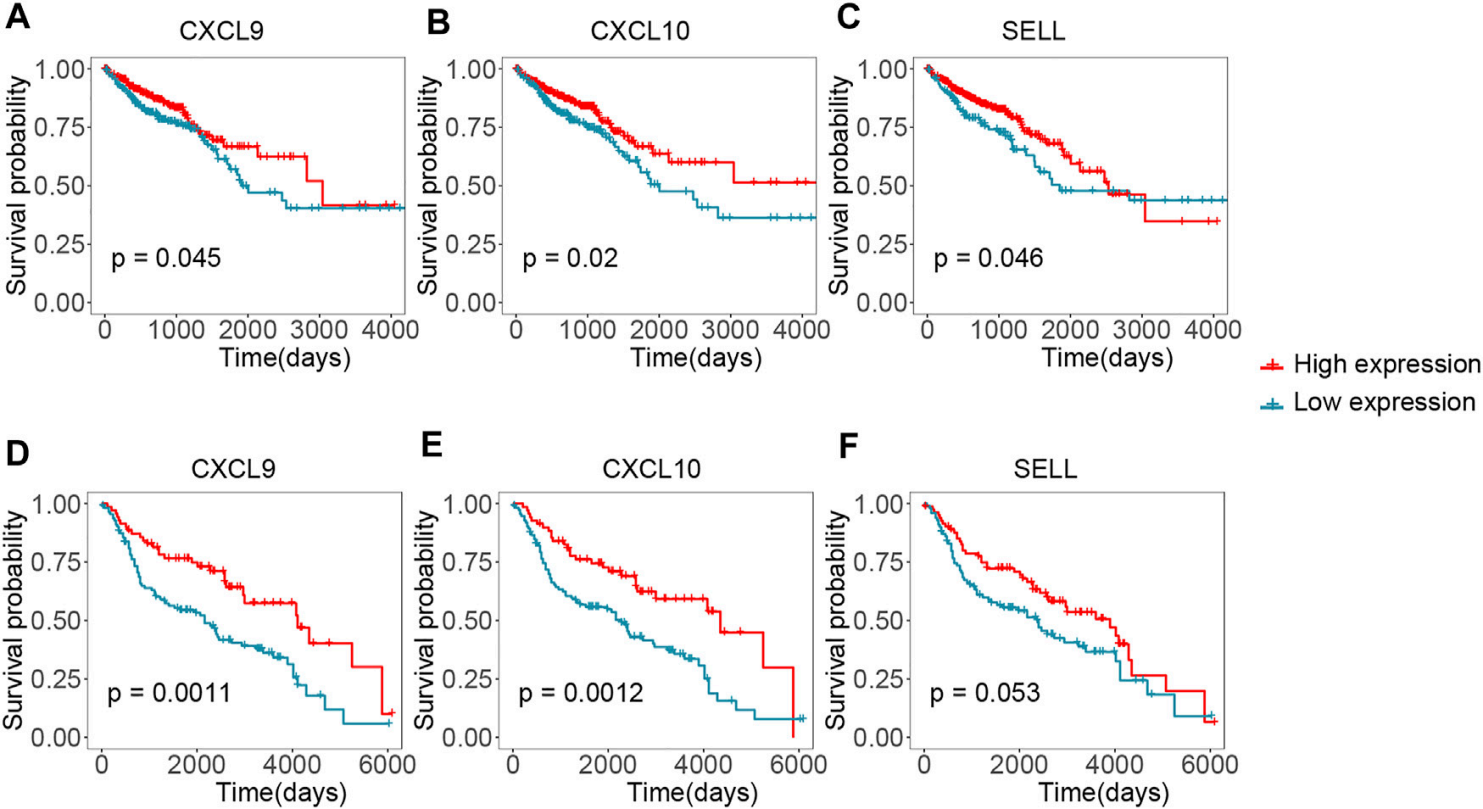
Validating the hub genes by survival time in TCGA and GEO cohorts. **(A–C)** Kaplan-Meier plots reflecting the overall survival status of CRC patients in TCGA cohort. **(D-F)** Kaplan-Meier plots showing the overall survival status of CRC patients in GSE41258 cohort.

We performed survival analysis between the 15 hub genes and the overall survival time to identify potential prognostic or predictive markers for CRC. Colorectal cancer samples were splited into high- and low- expression groups according to the optimal survival cut-off. We found that 11 hub genes were correlated with survival in TCGA dataset (Figures 6A–C and Supplementary Figure S3, p-value < 0.05, log-rank test). As shown in Figure 6, CXCL9 and CXCL10 were significantly correlated with the overall survival time in TCGA dataset (Figures 6A,B, p-value < 0.05, log-rank test), and a higher expression of them might correspond to better survival. Importantly, similar result was observed in the validation set GSE41258 (Figures 6D,E, p-value < 0.05, log-rank test). Moreover, high expression of SELL also showed longer overall survival in TCGA dataset (Figure 6C, p-value < 0.05, log-rank test), even though this pattern was not statistically significant in GSE41258 cohort (Figure 6F, p-value = 0.053, log-rank test). Higher expression of PTPRC and CCL4 had a better survival time in TCGA dataset (Supplementary Figure S3, *p*-value < 0.05, log-rank test) and showed a longer median survival time in GSE41258, but this correlation was not statistically significant (Supplementary Figure S4, 0.05 < *p*-value < 0.1, log-rank test).

## Discussion

Colorectal cancer is one of the most common pathological types of cancer. Previous research have demonstrated that tumor microenvironment play an important role in the occurrence and development of CRC (Peddareddigari et al., 2010; Kamal et al., 2020). Data from previous studies indicated that the infiltration of immune cells into the tumor bed may be a valuable prognostic factor in the treatment of colorectal tumor (Pagès et al., 2005; Galon et al., 2006; Galon et al., 2007; Ganesh et al., 2019). Research showed that the high density of infiltrating memory CD45RO+ T cells, one type of immune cell, was associated with the absence of signs of early tumor lymphovascular and perineural invasion, a less advanced tumor stage, and a good clinical outcome (Pagès et al., 2005). Cancer-associated fibroblasts (CAFs) are one of the most abundant and key components of the tumor mesenchyme among all the stromal cells (Liu et al., 2019). According to the study of Isella et al., the presence of high levels of CAFs was associated with poor prognosis in untreated CRC (Isella et al., 2015). Understanding the relationship between tumor microenvironment and patients’ clinical features is vital in figuring out cancer recurrence and metastasis mechanisms. However, this mechanism is not well-understood yet.

In this study, we used the ESTIMATE algorithm to evaluate the infiltration degree of immune and stromal cells in colorectal cancer. A total of 613 CRC patients were divided into two groups based on the immune and stromal scores calculated by the R function ESTIMATE. As a result, we found high immune score was related with prolonged survival time. This observation was in general agreement with the study of Mlecnik et al. (Mlecnik et al., 2016). Besides, we found lower stromal score indicated a longer overall survival time, which further confirmed previous work by Calon et al. (Calon et al., 2015). More importantly, when patients had high immune and low stromal scores, they displayed a significantly better clinical outcome. The similar trends were also observed in another independent dataset GSE41258. These results from our study may help elucidate the underlying mechanisms in colorectal cancer microenvironment and prognosis.

Apart from that, we found clinical factors including primary tumor location and tumor stage were significantly correlated with immune score in CRC. It is worth noting that right-sided colon cancer had significantly higher immune score, as compared to left-sided colon cancer or rectum cancer. These findings might explain why right-sided colon cancer, presenting a high level of neoantigens, responded well to immunotherapies rather than adjuvant chemotherapies (Ribic et al., 2003; Wang et al., 2015; Passardi et al., 2017; Baran et al., 2018). To the best of our knowledge, previous researches mainly focused on the difference between right-sided and left-sided colon cancer (Petrelli et al., 2017; Mao et al., 2018; Zhang et al., 2018). Our study provides a more comprehensive analysis about right-sided, left-sided, and rectum in CRC patients. Our results further indicated that immune infiltration was different among right, left, and rectal CRCs. These immune infiltrating differences might contribute to the different survival time of CRC patients and providing a potential explanation for prognostic survival associated with primary tumor location (Petrelli et al., 2017).

Through the immune and stromal scores related DEGs analysis, a total of 318 DEGs were screened out and many of them were involved in tumor microenvironment related biological processes and pathways. Specifically, based on the DEGs analysis and GSEA annotation results, 188 DEGs were significantly correlated with immune score and most of them were involved in function that modulates the frequency or extent of an immune system process. Based on the analysis of DEGs and annotation of GSEA, 150 genes were significantly correlated with stromal score and mainly enriched in a structure that lies outside the plasma membrane and surrounds the entire cells.

*Via* PPI network construction, 15 genes (CD86, ITGAM, PTPRC, FCGR3A, FCGR3B, MRC1, CD163, CCR2, SELL, CD69, CXCL10, CXCL8, CXCL9, CCL19 and CCL4) were selected as hub genes. Especially, three genes (CXCL9, CXCL10, and SELL) were detected to be correlated with overall survival time both in the TCGA dataset and the validation GEO dataset. As shown in Figure 6, their higher expression was associated with an increased survival rate, indicating that they might be potential prognostic targets of CRC.

C-X-C motif chemokine ligand 9 (CXCL9, also known as CMK and MIG) and C-X-C Motif Chemokine Ligand 10 (CXCL10, also known INP10 and SCYB10) are mainly involved in selective and non-covalent interaction with the CXCR3 chemokine receptor and cytokine activity according to the Gene Ontology annotation. The protein encoded by CXCL9 is a member of CXC chemokine family that participates in T cell trafficking. Previous study suggested that CXCL9 plays an important role in different types of tumors (Ding et al., 2016). CXCL9 can be a tumor suppressor in breast cancer, non‐small cell lung carcinoma, and colorectal cancer (Addison et al., 2000; Denkert et al., 2010; Wu et al., 2016). Conversely, it acts as tumor promoter in various types of cancer such as hepatocellular carcinoma, oral cavity squamous cell carcinoma, squamous cell cervical cancer, and chronic lymphocytic leukemia (Yan et al., 2011; Chang et al., 2013; Zhi et al., 2014; Liu et al., 2015). CXCL10 which is an important paralog of CXCL9, binds CXCR3 receptor to induce a variety of processes including chemotaxis, regulation of cell growth and apoptosis, regulation of angiostasis, and activation of immune cells (Liu et al., 2011; Sidahmed et al., 2012). The study of Chen et al. revealed that lower expression of CXCL10 was significantly associated with unsatisfied survival time (Chen et al., 2020). Our result showed that high expression of CXCL9 and CXCL10 were correlated with a better prognosis, which is consistent with studies of colorectal cancer in recent years (Wu et al., 2016; Chen et al., 2020).

SELL, also known as CD62L and L-selectin, belongs to the selectin family of glycoprotein adhesion molecules (Lefer, 2000), which is expressed on multiple tumor-infltrating immune cells and abundant in the surface of neutrophils (Lefer, 2000; Kumari et al., 2021). Recent study suggest that L-selectin might be a favorable prognosis factor in breast cancer (Kumari et al., 2021). To the best of our knowledge, there are limited studies about SELL expression and overall survival time in colorectal cancer. In this study, the high level of SELL was found correlated with better survival of CRC patients, indicating that SELL might be a new potential prognostic biomarker in CRC.

In Summary, based on the tumor immune and stromal analysis, we found that tumor microenvironment was related to CRC survival outcome and clinical characteristics such as tumor stage and location. And we identified a series of candidate genes which might serve as prognostic biomarkers in CRC. However, there were some limitations in our study. All analysis was based on public data mining instead of experiments. More experiments need to be carried out in order to further verify our conclusion and have a comprehensive insight on the potential link between the tumor microenvironment and colorectal cancer. Our current findings might provide insights into understanding the potential role of tumor microenvironment in CRC.

## Data Availability

Publicly available datasets were analyzed in this study. These data can be found in TCGA (https://portal.gdc.cancer.gov/) and GEO (https://www.ncbi.nlm.nih.gov/geo/).
