# Supplementary material for: Identifying Potential Biomarkers of Prognostic Value in Colorectal Cancer via Tumor Microenvironment Data Mining
Source: Front Genet. 2022 Feb 3;12:787208. doi: 10.3389/fgene.2021.787208 (PMC8890124; doi:10.3389/fgene.2021.787208)
Supplement: Supplementary file 1 [file DataSheet1.zip › Supplementary Figures.docx]

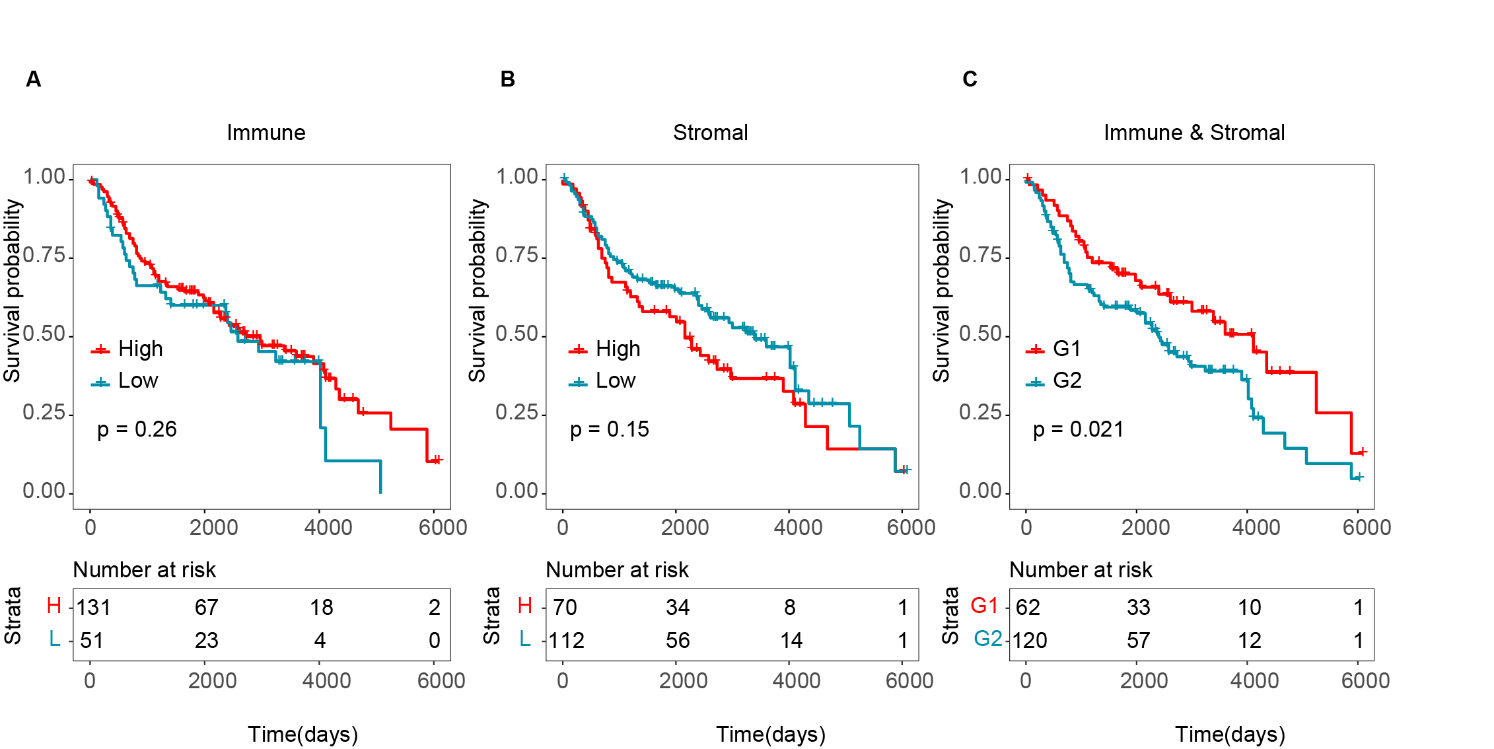
**Supplementary Figure S1.** Association between tumor microenvironment and overall survival time in GSE41258 CRC cohort. (A) Kaplan–Meier curves of high and low immune score groups. (B) Kaplan–Meier curves of high and low stromal score groups. (C) Kaplan–Meier curves of G1 (high immune score and low stromal score group) versus G2 (low immune and stromal score group, high immune and stromal score group, low immune score and high stromal score group).


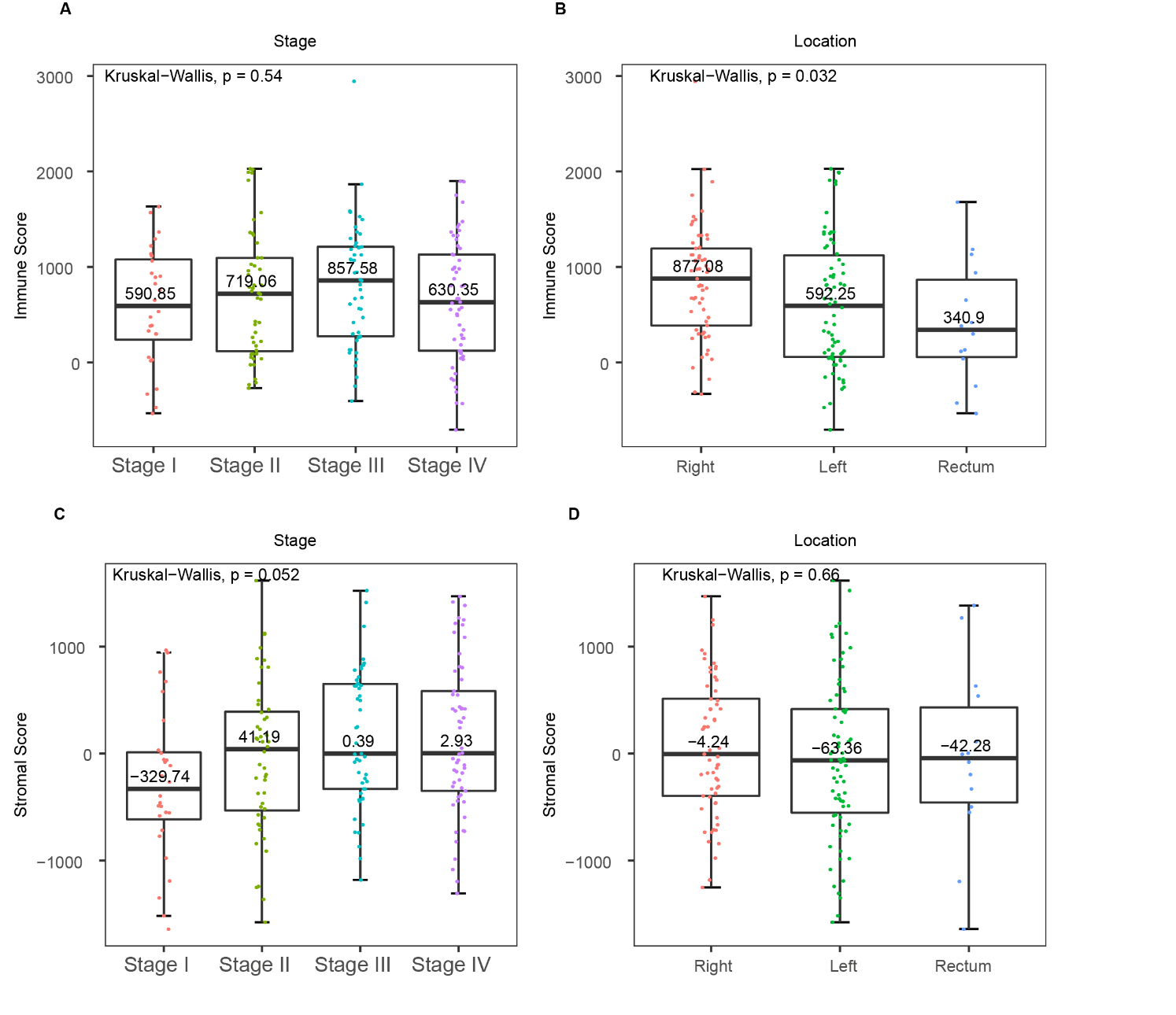


**Supplementary Figure S2.** Association between tumor microenvironment scores and clinical features in GSE41258 CRC cohort. (A) Distribution of immune scores in consecutive CRC tumor stages. (B) Distribution of immune scores from distinct CRC primary tumor locations. (C) Distribution of stromal scores in consecutive CRC tumor stages. (D) Distribution of stromal scores from different CRC primary tumor locations.


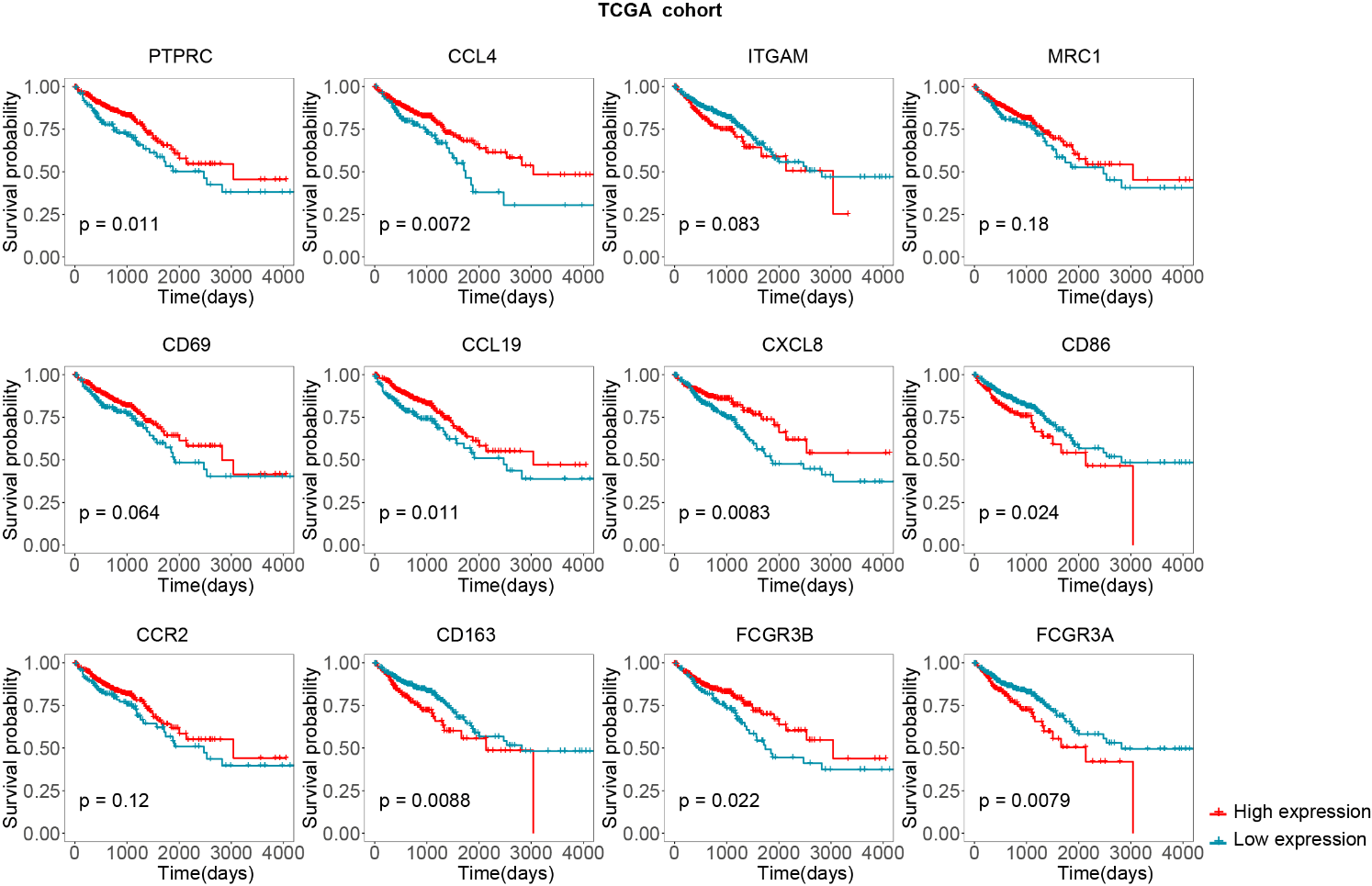


**Supplementary Figure S3.** Hub genes survival analysis in TCGA CRC dataset.


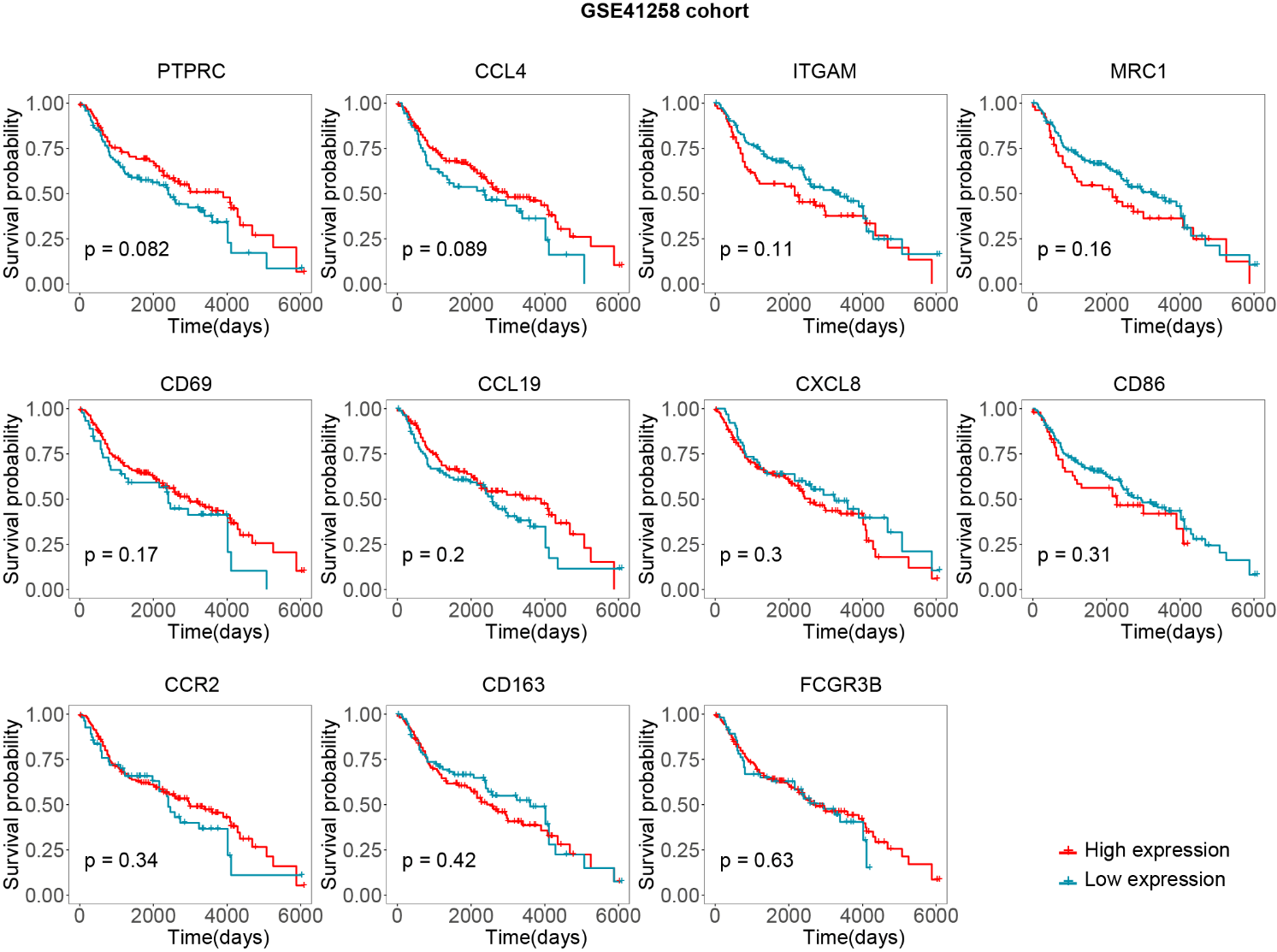


**Supplementary Figure S4.** Hub genes survival analysis in GSE41258 CRC dataset.
